# Model of the HVC neural network as a song motor in zebra finch

**DOI:** 10.3389/fncom.2024.1417558

**Published:** 2024-11-20

**Authors:** Pan Xia, Henry D. I. Abarbanel

**Affiliations:** Department of Physics, University of California, San Diego, La Jolla, CA, United States

**Keywords:** computational model, HVC, neuron interaction, song system, zebra finch

## Abstract

The nucleus HVC within the avian song system produces crystalized instructions which lead to precise, learned vocalization in zebra finches (*Taeniopygia guttata*). This paper proposes a model of the HVC neural network based on the physiological properties of individual HVC neurons, their synaptic interactions calibrated by experimental measurements, as well as the synaptic signal into this region which triggers song production. This neural network model comprises of two major neural populations in this area: neurons projecting to the nucleus RA and interneurons. Each single neuron model of HVC_RA_ is constructed with conductance-based ion currents of fast Na^+^ and K^+^ and a leak channel, while the interneuron model includes extra transient Ca^2+^ current and hyperpolarization-activated inward current. The synaptic dynamics is formed with simulated delivered neurotransmitter pulses from presynaptic cells and neurotransmitter receptor opening rates of postsynaptic neurons. We show that this network model qualitatively exhibits observed electrophysiological behaviors of neurons independent or in the network, as well as the importance of bidirectional interactions between the HVC_RA_ neuron and the HVC_I_ neuron. We also simulate the pulse input from A11 neuron group to HVC. This signal successfully suppresses the interneuron, which leads to sequential firing of projection neurons that matches measured burst onset, duration, and spike quantities during the zebra finch motif. The result provides a biophysically based model characterizing the dynamics and functions of the HVC neural network as a song motor, and offers a reference for synaptic coupling strength in the avian brain.

## 1 Introduction

Adult male zebra finches are extraordinary singers that produce highly crystallized and complex sequence of syllables during courtship (Bolhuis et al., 2010; Margoliash, 2010; Daou et al., 2013; Mooney, 1991; Mooney and Prather, 2005; Simonyan et al., 2012). Birdsong production from male zebra finches when they are directed toward females is an interesting model for studying complex vocal behavior. Birdsong and human speech share similar precisely integrated vocal and respiratory muscle activity, and have similar critical periods for vocal learning, which depends on early auditory experience and feedback (Doupe and Kuhl, 1999; Deregnaucourt et al., 2005; Mooney, 2009). Moreover, birds and humans share the same basic organizational features in their auditory periphery (Mooney, 2009). Therefore, modeling of the zebra finches’ song system can be very helpful to understand the mechanisms behind human audition and speech.

Studies of zebra finches have identified a specialized forebrain pathway that ultimately regulates syringeal and respiratory muscles to produce songs. Premotor nucleus HVC plays a critical role in singing and song learning (Fee and Scharff, 2010; Fee and Goldberg, 2011; Daou et al., 2013).

A subclass of HVC neurons (HVC_RA_ neurons) sends excitatory projections to the robust nucleus of arcopallium (RA), which in turn controls song acoustic features. During singing behavior, HVC_RA_ neurons fire short bursts of action potentials consistent across repeated renditions of the song (Kadakia et al., 2016; Hahnloser et al., 2002). An important hypothesis posits that the observed HVC_RA_ neurons’ bursts encode the temporal evolution of the birdsong (Long and Fee, 2008; Long et al., 2010; Lynch et al., 2016). Identifying how action potential bursts of HVC_RA_ neurons are generated and transferred from one cell to the next through the local neural network provides a foundation for understanding the generation of song timing information. This paper explores two major types of neurons in HVC: the HVC_RA_ projection neurons and the interneurons. HVC_RA_ neurons give rise to a descending song motor pathway required for song generation, while the inhibitory effect of HVC_I_ neurons is critical for modulating the activity of HVC_RA_ neurons (Long et al., 2010).Numerous intracellular recordings of HVC neurons have unveiled a variety of physiological properties and circuit mechanisms within the HVC (Daou et al., 2013; Daou and Margoliash, 2020; Mooney and Prather, 2005; Long et al., 2010), as well as the trigger input into HVC before the motif and neuron spikes during singing (Ben-Tov et al., 2023). There are also many HVC single neuron models focused on spike characteristics and different ion channels (Kadakia et al., 2016; Daou et al., 2013; Daou and Margoliash, 2020; Meliza et al., 2014; Breen et al., 2016), but less work has been done to reproduce the network activity (Li and Greenside, 2006; Long et al., 2010; GGA1; Armstrong and Abarbanel, 2016). Several earlier network models have successfully generated the series of HVC_RA_ neuron firing patterns. However, these models either proposed chain models without explaining the biophysical mechanism behind the series propagation, or failed to include electrical recording confirmed synaptic connections among various of neurons in the HVC (Li and Greenside, 2006; Gibb et al., 2009; Jin et al., 2007; Cannon et al., 2015; Armstrong and Abarbanel, 2016).

Here, we begin with conductance-based neuron models for individual HVC_RA_ and HVC_I_ cells. Each single-neuron model consists of ion channel dynamic equations verified by experiments, and both of them reproduce the spontaneous firing behavior of their corresponding neuron types under a background current (Daou et al., 2013; Armstrong and Abarbanel, 2016). Next, a microcircuit model is constructed with HVC_RA_ and HVC_I_ neurons based on experimentally established neurotransmitter pulses (Destexhe et al., 1994; Destexhe and Sejnowski, 2001), as well as recorded bidirectional synaptic interactions between them (Mooney and Prather, 2005). This microcircuit exhibits the basic neuron behavior when zebra finches are silent, and reproduces the sparse bursting patterns seen during female directed singing behavior once a model of dopaminergic innervation onto HVC from A11 neurons is included (Ben-Tov et al., 2023). Then, we extend the model microcircuit by adding more projection neurons along with homotypic synaptic interactions, and demonstrate that this framework successfully reproduces the time-locked firing pattern of excitatory HVC neurons during repeated renditions of zebra finches’ song discovered by Hahnloser et al. (2002). Most parameters in the single neuron models and synaptic current models are backed by experimental and simulation papers, and we discuss the model robustness under variation of the unknown or fine-tuned parameters.

## 2 Methods

### 2.1 Single neuron models

The basic units of our HVC neural network model are individual HVC_RA_ and HVC_I_ cells. The HVC_RA_ population projects to RA and gives rise to the song motor pathway (SMP). It plays a fundamental role in coordinating ensembles of neurons in RA, which in turn send motor commands to the brainstem for the precise control of the syringeal motor neurons and respiratory premotor neurons (Mooney, 2009; Mooney 2022). We also focus on the HVC_I_ neurons because prior works have shown that interneuron activity can modulate HVC_RA_ neurons’ firing and is important for birdsong (Armstrong and Abarbanel, 2016; Long et al., 2010).

Our neuron model is developed from conductance-based Hodgkin-Huxley-type neurons with sodium, potassium, and leak channels (Hodgkin and Huxley, 1952). The specific HVC_RA_ projecting neuron model is based on Kadakia et al. (2016) and Armstrong and Abarbanel (2016), as well as the electrophysiological recordings and simulations from Daou et al. (2013). Among the HVC_RA_ neuron channels, sodium and potassium currents produce fast-response spikes in response to stimulating currents, and leak current is a widely existing channel which is carried mainly by chloride and other ions. The model of inhibitory neurons (HVC_I_) is adapted from Breen et al. (2016), Armstrong and Abarbanel (2016) and Daou et al. (2013). Aside from the basic NaKL channels, the HVC_I_ cells are also shown to have a T-type low threshold calcium current (I_CaT_) and a hyperpolarization activated current (I_H_) (Breen et al., 2016; Armstrong and Abarbanel, 2016; Daou et al., 2013). The behavior of the calcium current is described by the Goldman–Hodgkin–Katz (GHK) equation to better reflect its current–voltage curve (Sterratt et al., 2011; Johnston and Samuel Miao-Sin, 1996). Compared to the classic Hodgkin–Huxley formulation, the GHK equation adds extra nonlinearity to the calcium channel (Bard Ermentrout and Terman, 2010).

The time evolutions of the cross-membrane voltages of the HVC_RA_ and HVC_I_ neurons are functions of the currents that flow across ion channels specific for certain types of neurons, as well as synaptic interactions and background stimulus current. All these components can be summarized in the following equations:

RA projection neuron:


$$C\frac{dV_{RA}\left(t\right)}{dt}=I_{Na}\left(t\right)+I_{K}\left(t\right)+I_{L}\left(t\right)+\sum I_{syn}\left(t\right)+I_{\mathrm{background}}$$

Interneuron:


$$\begin{array}{l} C\frac{dV_{I}\left(t\right)}{dt}=I_{Na}\left(t\right)+I_{K}\left(t\right)+I_{L}\left(t\right)+I_{\mathrm{CaT}}\left(t\right)+I_{H}\left(t\right)\\ +\sum I_{syn}\left(t\right)+I_{\mathrm{background}} \end{array}$$

Here, C is the membrane capacitance. $V_{RA}\left(t\right)$ and $V_{I}\left(t\right)$ are the membrane potentials of HVC_RA_ and HVC_I_ neuron, respectively. Sodium, potassium, leak, low threshold calcium, and hyperpolarization activated currents are represented by I_type_, i.e., I_Na_, I_K_, I_L_, I_CaT_, and I_H_, respectively. The summation of the $I_{syn}\left(t\right)$ terms represents all the synaptic input currents from both inside and outside HVC. $I_{\mathrm{background}}$ refers to the ambient background stimulus which is usually a DC current. Each ion channel current can be expressed as a function of voltage V(t) and gating variables $G_{i}\left(t\right)$ = [ $m\left(t\right)$ , $h\left(t\right)$ , $n\left(t\right)$ , $a\left(t\right)$ , $b\left(t\right)$ , $H\left(t\right)$ ] (Johnston and Samuel Miao-Sin, 1996, Daou et al., 2013, Kadakia et al., 2016, Armstrong and Abarbanel, 2016), illustrated in the following equations:


$$I_{Na}\left(t\right)=g_{Na}m{\left(t\right)}^{3}h\left(t\right)\left(E_{Na}-V\left(t\right)\right)$$

$$I_{K}\left(t\right)=g_{K}n{\left(t\right)}^{4}\left(E_{K}-V\left(t\right)\right)$$

$$I_{L}\left(t\right)=g_{L}\left(E_{L}-V\left(t\right)\right)$$

$$I_{\mathrm{CaT}}\left(t\right)=g_{\mathrm{CaT}}\cdot a{\left(t\right)}^{3}b{\left(t\right)}^{3}\cdot GHK\left(V\left(t\right),Ca\left(t\right)\right)$$

$$I_{H}\left(t\right)=g_{H}H{\left(t\right)}^{2}\left(E_{H}-V\left(t\right)\right)$$

With the definition of $GHK\left(V\left(t\right),Ca\left(t\right)\right)$ written as:


$$GHK\left(V\left(t\right)\right)=V\left(t\right)\cdot \frac{{\left[Ca\right]}_{ext}\exp\left(-ZFV\left(t\right)/RT\right)-\left[Ca\right]\left(t\right)}{1-\exp\left(-ZFV\left(t\right)/RT\right)}$$

In the ion current equations, all parameters denoted as “ g ” are the maximum conductances of corresponding ion channels. The parameters named as “ E ” are the respective reversal potentials. In the GHK equation, ${\left[Ca\right]}_{ext}$ is the constant extracellular concentration of calcium ions, and $\left[Ca\right]$ is the intracellular calcium concentration evolving with time. Z is the valence of calcium ions. F is the Faraday constant and R is the gas constant. T represents the temperature which is 310 K in our case. All the gating variables $G_{i}\left(t\right)$ = [ $m\left(t\right)$ , $h\left(t\right)$ , $n\left(t\right)$ , $a\left(t\right)$ , $b\left(t\right)$ , $H\left(t\right)$ ] obey a similar set of equations (Johnston and Samuel Miao-Sin, 1996, Daou et al., 2013, Kadakia et al., 2016, Armstrong and Abarbanel, 2016):


$$\frac{dG_{i}\left(t\right)}{dt}=\frac{\eta_{Gi}\left(V\left(t\right)\right)-G_{i}\left(t\right)}{\tau_{Gi}\left(V\left(t\right)\right)}$$

$$\eta_{Gi}\left(V\left(t\right)\right)=\frac{1}{2}+\frac{1}{2}\tanh\left(\frac{V\left(t\right)-V_{Gi}}{\Delta V_{Gi}}\right)$$

$$\tau_{Gi}\left(V\left(t\right)\right)=\tau_{Gi0}+\tau_{Gi1}\left[1-\tanh^{2}\left(\frac{V\left(t\right)-V_{Gi}}{\Delta V_{Gi}}\right)\right]$$

Here, $V_{Gi}$ , $\Delta V_{Gi}$ , $\tau_{Gi0}$ and $\tau_{Gi1}$ are parameters for their corresponding gating variable $G_{i}\left(t\right)$ . The dynamics of $H\left(t\right)$ is the only exception here: $\eta_{H}\left(V\left(t\right)\right)$ and $\tau_{H}\left(V\left(t\right)\right)$ use different values of $\Delta V_{H}$ . The intracellular calcium concentration is also a function of time:


$$\frac{d\left[Ca\right]\left(t\right)}{dt}=\phi I_{\mathrm{CaT}}+\frac{Ca_{0}-\left[Ca\right]\left(t\right)}{\tau_{Ca}}$$

where the parameter $Ca_{0}$ is the intracellular calcium concentration during equilibrium state. All the values for the HVC_RA_ neuron model parameters are listed in Table 1; corresponding values for the HVC_I_ cell can be found in Table 2. The parameters governing the dynamics of gating variables [ $m\left(t\right)$ , $h\left(t\right)$ , $n\left(t\right)$ ] and the parameters [ $E_{Na},E_{K},E_{L},C$ ] have the same set of values for both the HVC_RA_ neuron model and the interneuron model, which are listed in Table 1.

**Table 1:** Parameter values for HVC_RA_ projecting neurons.

Parameter	Value	Reference	Parameter	Value	Reference
$g_{Na}$	1,050 nS	Kadakia et al. (2016)	$V_{h}$	−45 mV	Kadakia et al. (2016)
$E_{Na}$	55 mV	Kadakia et al. (2016)	$\Delta V_{h}$	−7 mV	Kadakia et al. (2016)
$g_{K}$	120 nS	Kadakia et al. (2016)	$\tau_{h0}$	0.1 ms	Kadakia et al. (2016)
$E_{K}$	−90 mV	Kadakia et al. (2016)	$\tau_{h1}$	0.75 ms	Kadakia et al. (2016)
$g_{L}$	3 nS	Kadakia et al. (2016)	$V_{n}$	−35 mV	Kadakia et al. (2016)
$E_{L}$	−80 mV	Kadakia et al. (2016)	$\Delta V_{n}$	10 mV	Kadakia et al. (2016)
$V_{m}$	−30 mV	Kadakia et al. (2016)	$\tau_{n0}$	0.1 ms	Kadakia et al. (2016)
$\Delta V_{m}$	9.5 mV	Kadakia et al. (2016)	$\tau_{n1}$	0.5 ms	Kadakia et al. (2016)
$\tau_{m0}$	0.01 ms	Kadakia et al. (2016)	C	10 pF	Armstrong and Abarbanel (2016)
$\tau_{m1}$	0.0 ms	Kadakia et al. (2016)			

Kadakia et al. (2016) constructed an HVC_RA_ model with a particular choice of parameters, which reproduced the neuron response with respect to pseudo-noisy dendritic currents. The HVC_RA_ neuron model described in this paper is a simplified version of the one in Kadakia et al. (2016) and the simulated HVC_RA_ model proposed by Armstrong and Abarbanel (2016). Units: mV, millivolts; ms, milliseconds; pF, pico-Farads; nS, nano-Siemens.

**Table 2:** Parameter values for interneuron.

Parameter	Value	Reference	Parameter	Value	Reference
$g_{Na}$	1,200 nS	Armstrong and Abarbanel (2016)	$\Delta V_{a}$	32.9 mV	Breen et al. (2016)
$g_{K}$	200 nS	Armstrong and Abarbanel (2016)	$\tau_{a0}$	4.44 ms	Breen et al. (2016)
$g_{L}$	3 nS	Armstrong and Abarbanel (2016)	$\tau_{a1}$	4.24 ms	Breen et al. (2016)
$g_{H}$	2 nS	Armstrong and Abarbanel (2016)	$V_{b}$	−62 mV	Breen et al. (2016)
$E_{H}$	−40 mV	Armstrong and Abarbanel (2016)	$\Delta V_{b}$	−62.5 mV	Breen et al. (2016)
$V_{H}$	−60 mV	Armstrong and Abarbanel (2016)	$\tau_{b0}$	2.9 ms	Breen et al. (2016)
$\Delta V_{H}for\;\eta$	−10 mV	Armstrong and Abarbanel (2016)	$\tau_{b1}$	7.57 ms	Breen et al. (2016)
$\Delta V_{H}for\;\tau$	−5.5 mV	Armstrong and Abarbanel (2016)	${\left[Ca\right]}_{ext}$	2,500 μM	Breen et al. (2016)
$\tau_{H0}$	214 ms	Armstrong and Abarbanel (2016)	$Ca_{0}$	1.11 μM	Breen et al. (2016)
$\tau_{H1}$	158 ms	Armstrong and Abarbanel (2016)	ϕ	3.88 μM/(ms·pA)	Breen et al. (2016)
$g_{\mathrm{CaT}}$	0.1 nS	Armstrong and Abarbanel (2016)	$\tau_{Ca}$	0.143 ms	Breen et al. (2016)
$V_{a}$	−30 mV	Armstrong and Abarbanel (2016)			

Breen et al. (2016) estimated the parameter values using a voltage recording of a real interneuron in vitro. Values are chosen based on modeling of HVC neurons in Breen et al. (2016), Armstrong and Abarbanel (2016), and Kadakia et al. (2016). The parameter values for [ $E_{Na},E_{K},E_{L},C,\;V_{m},\;\Delta V_{m},\;\tau_{m0},\;\tau_{m1},\;V_{h},\;\Delta V_{h},\;\tau_{h0},\;\tau_{h1},\;V_{n},\;\Delta V_{n},\;\tau_{n0},\;\tau_{n1}$ ] can be found in Table 1. Units: mV, millivolts; ms, milliseconds; nS, nano-Siemens; μM, micro-molar; pA, pico-Amp.

### 2.2 Synapses

The synaptic dynamics is built on the formalism of neurotransmitter pulses and the fraction of opening neurotransmitter acceptors, based on the data from Destexhe and Sejnowski (2001) and Destexhe et al. (1994). For presynaptic neurotransmitter release, assuming that all intervening reactions in the release process are fast and can be considered at steady state, the neurotransmitter concentration [T] can be expressed as:


$$\left[T\right]=\frac{{\left[T\right]}_{\text{max}}}{1+\exp\left[-\left(V_{pre}-V_{p}\right)/K_{p}\right]}$$

where ${\left[T\right]}_{\text{max}}$ is the maximal concentration of neurotransmitters in the synaptic cleft. $V_{pre}$ is the presynaptic cell voltage. $K_{p}$ is the steepness and $V_{pre}$ sets the value of which the function is half activated. This is a simplified model of the neurotransmitter release process compared to a kinetic model involving calcium diffusion and gradients, which introduces a smoother transformation between presynaptic voltage and neurotransmitter concentration.

Postsynaptic neurotransmitter receptors have several different types, each with specific response to the same concentration of corresponding neurotransmitters. Previous studies have confirmed that the local axon collaterals of HVC_RA_ neurons release glutamate, and excite interneurons by activating ionotropic glutamate receptors of the *α*-amino-3-hydroxy-5-methyl-4-isoxazolepropionic acid (AMPA) subtype (Mooney and Prather, 2005; Colquitt et al., 2021). For the inhibitory connections from interneurons to HVC_RA_ cells, this fast hyper-polarizing response is mediated by *γ*-aminobutyric acid (GABA) and GABA_A_ type receptors (Mooney and Prather, 2005; Colquitt et al., 2021). Under the assumption that these two types of neurotransmitters both bind to the receptors at a constant rate, the postsynaptic kinetics can be described by the following set of equations:


$$\frac{dr}{dt}=\alpha_{\mathrm{AMPA}/\mathrm{GABA}}\left[T\right]\left(1-r\right)-\beta_{\mathrm{AMPA}/\mathrm{GABA}}r$$

$$I_{ij}=g_{ij}r_{j}\left(V_{j}\left(t\right)-E_{\mathrm{AMPA}/\mathrm{GABA}}\right)$$

where r is the fraction of the postsynaptic receptors in the open state. Its dynamics depends on $\alpha_{\mathrm{AMPA}/\mathrm{GABA}}$ , the gate opening rate, and $\beta_{\mathrm{AMPA}/\mathrm{GABA}}$ , the gate closing rate. They take different values for AMPA and GABA_A_ type receptors. $I_{ij}$ is the current seen by postsynaptic cell *j* as a result of input from presynaptic neuron *i*. $g_{ij}$ is the maximal conductance and $E_{\mathrm{AMPA}/\mathrm{GABA}}$ is the synaptic reversal potential. $V_{j}\left(t\right)$ is the instantaneous membrane voltage of the postsynaptic cell. Parameter values for synaptic dynamics can be found in Table 3.

**Table 3:** Parameter values for synaptic interactions.

Parameter	Value	Reference	Parameter	Value	Reference
$g_{A11,\;INT}$	8 nS	Destexhe and Sejnowski (2001) and Hiratani and Fukai (2018)	$\alpha_{\mathrm{GABA}}$	5 /(mM·ms)	Destexhe and Sejnowski (2001)
$g_{INT,RA}$	8 nS	Destexhe and Sejnowski (2001) and Hiratani and Fukai (2018)	$\alpha_{\mathrm{AMPA}}$	1.1 /(mM·ms)	Destexhe and Sejnowski (2001)
$g_{RA,\;INT}$	7 nS	Destexhe and Sejnowski (2001) and Hiratani and Fukai (2018)	$\beta_{\mathrm{GABA}}$	0.18 /ms	Destexhe and Sejnowski (2001)
$g_{RA,RA}$	10 or 8.2 nS	*	$\beta_{\mathrm{AMPA}}$	0.19 /ms	Destexhe and Sejnowski (2001)
$E_{\mathrm{GABA}}$	−80 mV	Destexhe and Sejnowski (2001)	$K_{p}$	5 mV	Destexhe and Sejnowski (2001)
$E_{\mathrm{AMPA}}$	0 mV	Destexhe and Sejnowski (2001)	$V_{p}$	2 mV	Destexhe and Sejnowski (2001)
${\left[T\right]}_{\text{max}}$	2.84 mM	Destexhe et al. (1994)			

*Means the value has been tuned. Values from Destexhe and Sejnowski (2001) are obtained from the best fit of the synaptic kinetic equations to recorded AMPA/GABA currents. Units: mV, millivolts; ms, milliseconds; nS, nano-Siemens; mM, milli-molar.

The value of maximal conductance $g_{ij}$ of the synaptic current between two neurons is obtained by two factors: the number of synapses connecting neuron i and neuron j, and the maximal conductance for a single synapse. Previous morphological studies show that there are usually multiple synaptic connections between two connected neurons in different cortical circuits across the brain (Hiratani and Fukai, 2018). More specifically, the average number of synapses per connection is estimated to be around 10 in the barrel cortex (Hiratani and Fukai, 2018). For inhibitory interactions, estimation for the maximal conductance of a single GABAergic synapse with GABA_A_ type currents is in the range of 0.25 to 1.2 nS (Ropert et al., 1990; De Koninck and Mody, 1994). Therefore, we take the median value of 0.8 nS, so the maximal conductance for inhibitory connections between two neurons is estimated to be around 8 nS. For excitatory synaptic interactions, measurements of miniature synaptic currents and analysis estimate that the maximal conductance of AMPA-mediated is between 0.35–1.0 nS in the neocortical and hippocampus pyramidal cells (Stricker et al., 1996; Burgard and Hablitz, 1993; McBain and Dingledine, 1992). Thus, $g_{RA,\;INT}$ , the maximal conductance from an excitatory HVC_RA_ neuron to the postsynaptic HVC_I_ neuron is set to 7 nS in our modeling. The only parameter we vary is the maximal conductance from one HVC_RA_ neuron to another, i.e., $g_{RA,RA}\text{.}$ This synaptic connection strength for homotypic HVC_RA_ cell pairs is assigned a higher value to ensure the excitatory input is large enough to awaken the postsynaptic HVC_RA_ neuron. There will be more discussion about this fine-tuned parameter value in the *Results* section.

### 2.3 Trigger signal

When male zebra finches sing during courtship, HVC activity is closely synchronized with song production. To enable directed song production, a neural circuit receives information about sexual motivations and then communicates with the HVC neural network to start the sequence of stereotyped syllables. A11 cells are part of this neural circuit, which connect to HVC to gate the song motif (Ben-Tov et al., 2023).

The midbrain A11 cell group is implicated in motor control, motivation, and reproduction (Mohebi et al., 2019; da Silva et al., 2018). A11 neurons in songbirds receive sexual motivation input from the medial preoptic nucleus (POM) (Riters and Alger, 2004), and project axons into HVC amongst other regions. A11 neurons and their axons in HVC are crucial for female-directed singing. Male zebra finches with lesioned A11 cell bodies or A11 terminals in HVC failed to sing when presented with a female bird (Ben-Tov et al., 2023). We sought to simulate the physiological changes in HVC neural network following activation of A11-HVC projection.

**Figure 1:**
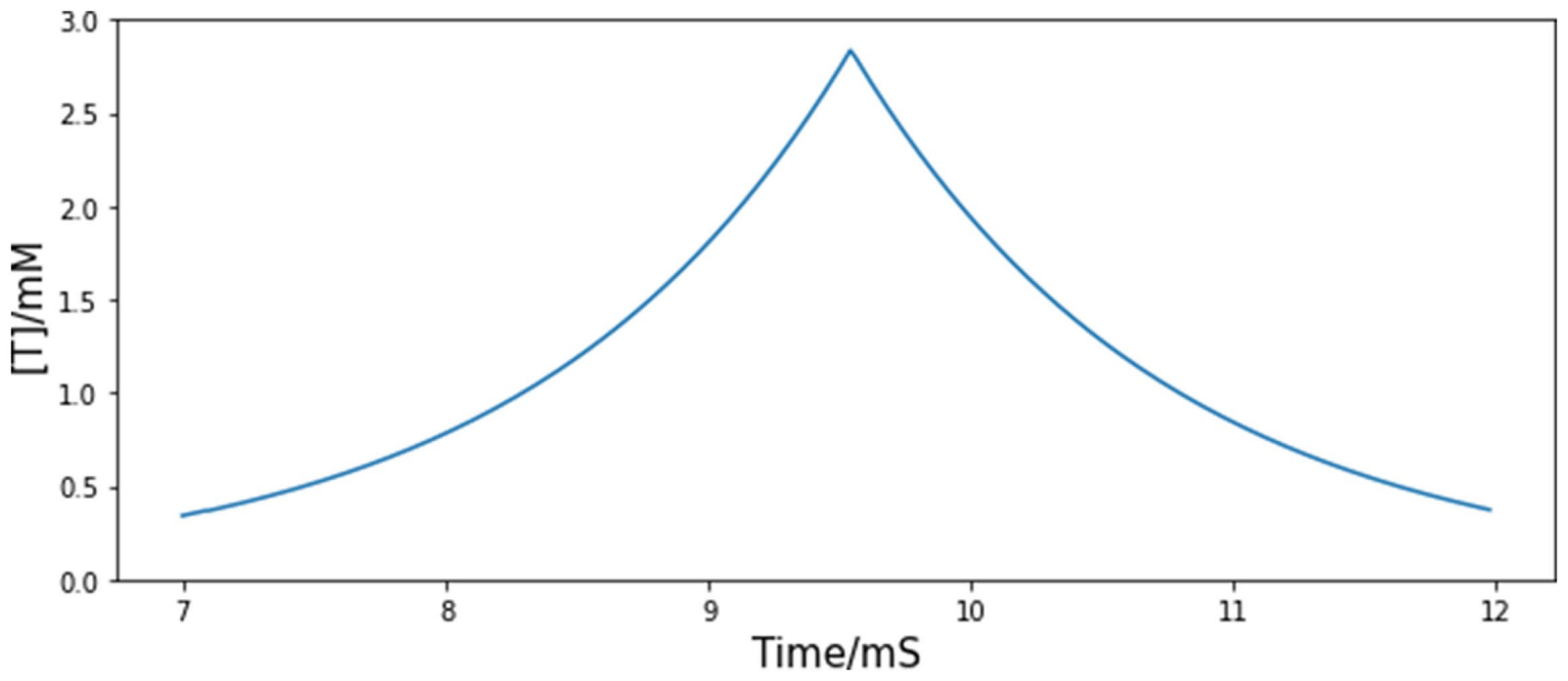
Simulated time course of injected neurotransmitter concentration [T] from A11 axons to HVC. The shape of the simulated trajectory of [T] is similar to the GCaMP recordings from A11 axons in HVC during female directed song motifs (see Figure 6F, Ben-Tov et al., 2023), but the timescale is determined to match the measured time course of neurotransmitters in the synaptic cleft since GCaMP signal has a large time lag.

During *in vivo* experiment, fiber photometry reveals that the GCaMP signal of A11 axons in HVC first rapidly increases during the introductory notes (repetitive call-like vocalizations that immediately precede the song motif), reaches the peak point at the motif onset, and then decreases at an almost constant speed (Figure 1). By assuming that the trajectory of the neurotransmitter concentration in the synaptic cleft in HVC is similar to the shape of the measured calcium signal, we can approximate the dynamics of neurotransmitter concentration from A11 axons with the following equations:


$$\left[T\right]\left(t\right)={\left[T\right]}_{\text{min}}\;\left(t<0\right)$$

$$\left[T\right]\left(t\right)={\left[T\right]}_{\text{min}}e^{t/\tau_{r}}\;\left(0<t<t_{\text{max}}\right)$$

$$\left[T\right]\left(t\right)=T_{\text{max}}e^{-t/\tau_{f}}+{\left[T\right]}_{\text{min}}\;\left(t>t_{\text{max}}\right)$$

Again, $\left[T\right]\left(t\right)$ is the neurotransmitter concentration as a function of time. ${\left[T\right]}_{\text{min}}$ represents the baseline concentration, i.e., [T] before the trigger signal arrives. $\tau_{r}$ and $\tau_{f}$ are the time constants which determine the rate of rise and fall for neurotransmitters, respectively. $t_{\text{max}}$ means the time point when the concentration transits from rise to fall. For $T_{\text{max}}$ , it is a constant chosen to ensure the continuity of neurotransmitter concentration at time $t_{\text{max}}$ . Therefore, the value of $T_{\text{max}}$ is entirely determined by other parameters:


$$T_{\text{max}}={\left[T\right]}_{\text{min}}\left(e^{t_{\text{max}}/\tau_{r}}-1\right)\times e^{t/\tau_{f}}$$

Assuming that the maximum neurotransmitter concentration is ${\left[T\right]}_{\text{max}}$ , the value of the transition time can be derived from previous equations:


$$t_{\text{max}}=\log\left(\frac{{\left[T\right]}_{\text{max}}}{{\left[T\right]}_{\text{min}}}\right)\times \tau_{r}$$

The values of all parameters related to the A11 neurotransmitter dynamics are listed in Table 4. The time course of the trigger signal neurotransmitter concentration is displayed in Figure 1. We choose ${\left[T\right]}_{\text{max}}$ to be 2.84 mM, a value which corresponds to the observation of maximal transmitter concentration in Destexhe et al. (1994). ${\left[T\right]}_{\text{min}}$ is chosen to be positive so that the value of $\left[T\right]\left(t\right)$ is not constantly zero, and it is set to a small value so that the A11 stimulus does not affect HVC neural network outside the motif onset period. Other than these two restrictions, the exact value of ${\left[T\right]}_{\text{min}}$ does not make a big difference to the modeling result (see *Results* section for more details about this parameter). The rise and fall timescales for the recorded GCaMP signal are up to 1 s, but we do not use this to determine the values of $\tau_{r}$ or $\tau_{f}$ . The reason is that GCaMP recordings have a large time lag compared to real neuron activities, whose value could be up to a few seconds (Storace et al., 2015). The fall time constant is set to 1.2 ms, same as the measured decay time course of free neurotransmitters in the synaptic cleft of cultured hippocampal synapses (Clements et al., 1992), and within the normally estimated decay time range (Scimemi and Beato, 2009). The rise time constant is chosen to match it so that the trajectory of neurotransmitter concentration is symmetric. Based on the above choices of parameter values, the combined time span of rise and fall is approximately 5 ms (see Figure 1). The postsynaptic kinetics of the A11-HVC projection can be described with the same equations in Section 2 Synapses.

**Table 4:** Parameter values for triggering.

Parameter	Value	Reference	Parameter	Value	Reference
${\left[T\right]}_{\text{min}}$	0.001 mM	*	$\tau_{r}$	1.2 ms	Clements et al. (1992) and Ben-Tov et al. (2023)
${\left[T\right]}_{\text{max}}$	2.84 mM	Destexhe et al. (1994)	$\tau_{f}$	1.2 ms	Clements et al. (1992)

*Means the value has been tuned. See text for details. Units: ms, milliseconds; mM, milli-molar.

### 2.4 Simulation

For all the voltage and current time series shown in this paper, the dynamical equations were written in Python, and the results were integrated with Python’s adaptive fourth order Runge–Kutta “odeINT” using a step size of 0.02 ms. A smaller step size did not lead to different results.

## 3 Results

This section illustrates, via the time course of cross-membrane voltages of two types of neuron models, how they function independently, respond to external stimulus, and coordinate within the network to reproduce important experimental observations. We also test the importance of various experimental established synaptic currents by adding them to the modeled network one by one, and explore model robustness at the end of this section.

### 3.1 Behavior of single neuron model

**Figure 2:**
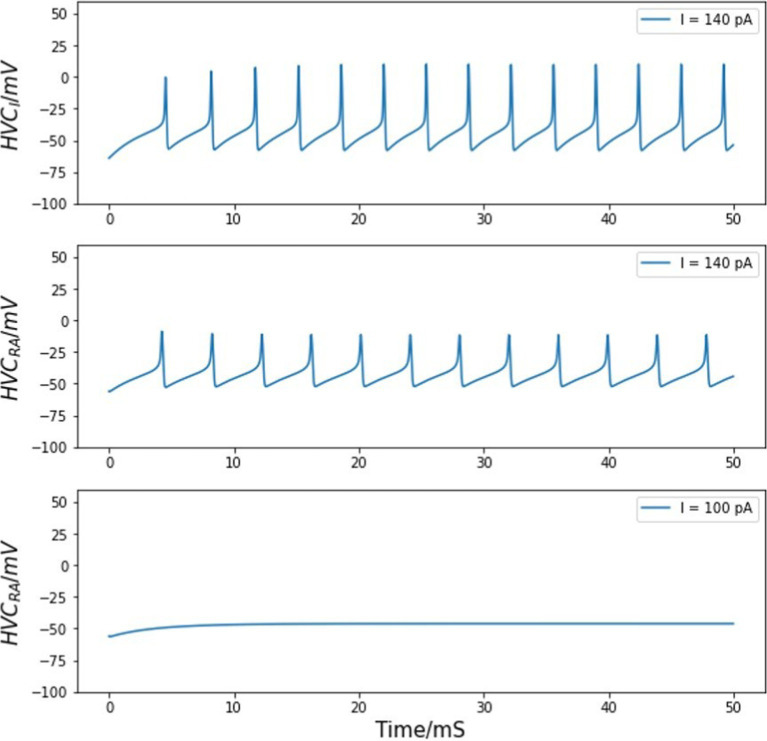
Voltage traces of HVC neurons. Top panel: voltage of an HVC interneuron neuron in response to a background current. Middle panel: an HVC_RA_ neuron exhibits action potential given a threshold stimulus of about 140 pA. Bottom panel: the HVC_RA_ model stays silent under a lower background current of 100 pA.

With the published set of parameters shown in Tables 1, 2, the two models reproduce qualitative features of HVC_RA_ and HVC_I_ neurons observed in whole-cell patch clamp experiments (Daou et al., 2013). For the excitatory neurons, although an HVC_RA_ neuron *in vivo* usually generates a single burst synchronized with singing, those projecting neurons will no longer fire once per song, but rather multiple times without inhibitory neurons (Long et al., 2010). Without inhibition input from any HVC_I_ neurons, HVC_RA_ cells can fire with a background stimulus above the threshold of about 100 pA (Daou et al., 2013). Our simulations reproduce this behavior with a threshold of about 140 pA. Figure 2 shows the membrane voltage of one independent HVC_RA_ neuron given an injected current of this threshold stimulus. For the interneuron, Figure 2 shows the stereotyped firing of the interneuron model under the same injected current.

### 3.2 Trigger signal into HVC

The midbrain A11 cell group is implicated in motor control, motivation, and reproduction (Mohebi et al., 2019; da Silva et al., 2018). A11 neurons in songbirds receive sexual motivation input from the medial preoptic nucleus (POM) (Riters and Alger, 2004), and project axons into HVC amongst other regions. A11 neurons and their axons in HVC are crucial for female-directed singing. Male zebra finches with lesioned A11 cell bodies or A11 terminals in HVC failed to sing when presented with a female bird (Ben-Tov et al., 2023). We sought to simulate the physiological changes in HVC neural network following activation of A11-HVC projection.

The A11 cell group is thought to distribute information about sexual motivation to HVC. The activity of A11 terminals in HVC starts to increase above the baseline before the first syllable as shown in Figure 1, which may serve as a trigger for motif initiation (Ben-Tov et al., 2023).

To simulate HVC neuron activities after the trigger signal arrives, we first expose an interneuron to the neurotransmitter pulses. We choose interneuron instead of HVC_RA_ neuron because those projecting neurons fail to fire at a particular temporal location during each motif without the presence of HVC_I_ cells (Kosche et al., 2015; Armstrong and Abarbanel, 2016). Therefore, there is a high probability that the interneurons receive the signal from A11 cell group and then coordinate the behavior of HVC_RA_ neurons.

**Figure 3:**
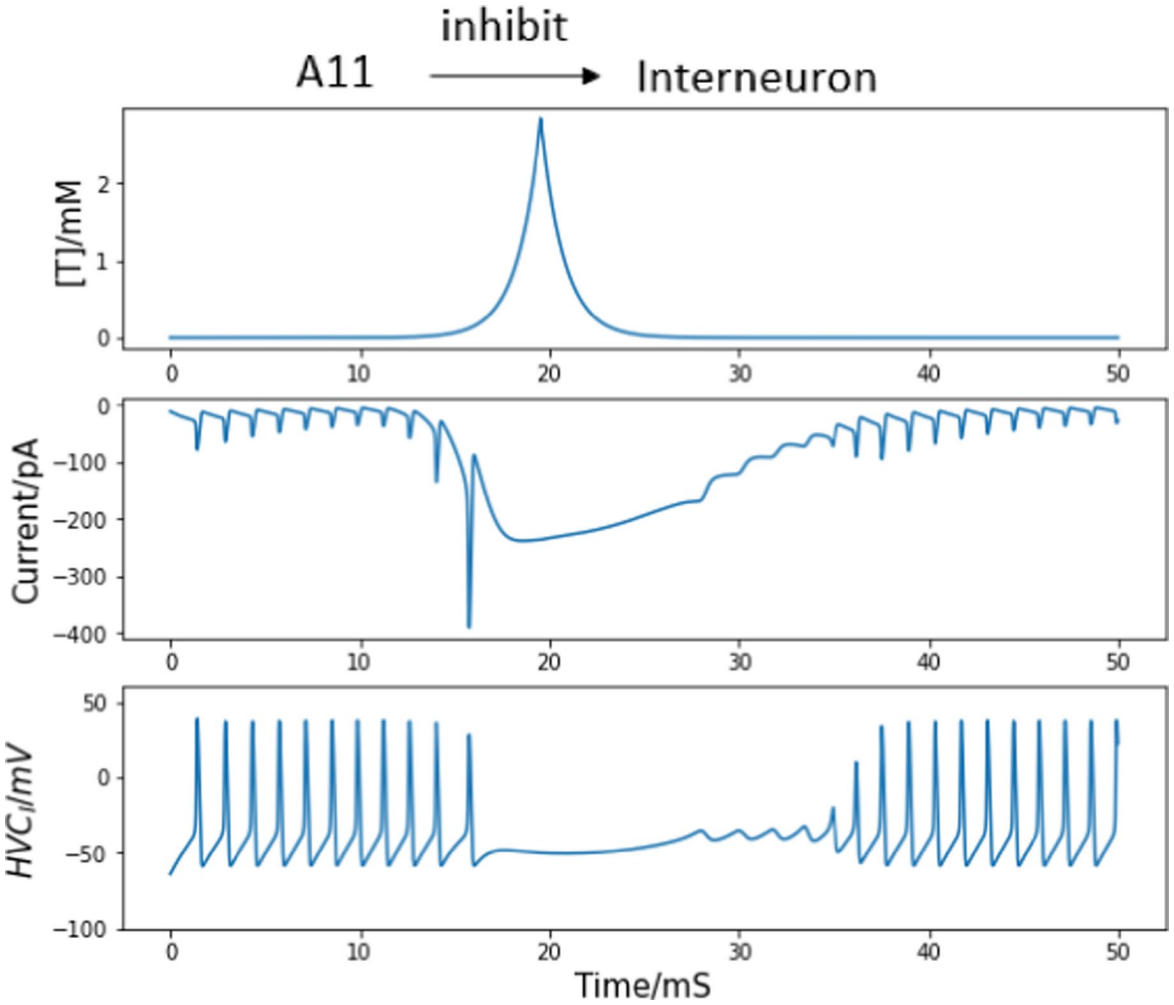
HVC_I_ neuron responses to the trigger current. The trigger signal happens at 10 ms. Top panel: synaptic connections between A11 axons and interneuron. Middle top: Simulated trajectory of injected neurotransmitter concentration [T] from A11 axons to HVC. Middle bottom: postsynaptic current from A11 cell axons to interneuron corresponding to the neurotransmitter concentration path in the middle top panel. Bottom panel: membrane voltage of the interneuron. The continuous firing of HVC_I_ neurons is interrupted by the inhibition input arising from 10 ms, corresponding to the intermittent silence throughout singing.

Normal and uninterrupted singing consists of a fixed sequence of syllables, which are interspaced by brief inhalation gaps. Both the syllables and the gaps occur in a fixed chronological order, and they are precisely timed during repeated renditions of the same motif. During this process, single HVC_I_ neuron’s recordings show relatively sustained firing throughout the song with intermittent gaps (Armstrong and Abarbanel, 2016). However, each HVC_RA_ neuron is observed to only burst once throughout a motif at a specific time. Together with the fact that HVC_RA_ neurons fail to fire at a particular temporal location during each motif without the presence of interneurons (Kosche et al., 2015; Armstrong and Abarbanel, 2016), we can assume the input from A11 axons to interneurons to be inhibitory, which stops HVC_I_ neurons from continually firing. Following the postsynaptic current equations, the inhibitory current corresponding to A11 neurotransmitters and the response of single HVC_I_ neuron are depicted in Figure 3. The trigger signal is not present until 10 ms so that the interneuron voltages before and after the motif onset are both revealed.

### 3.3 Interactions between HVC_I_ and HVC_RA_ neuron

Since the axonal and dendritic processes from all major types of HVC neurons as well as axons from HVC afferents are interwoven with each other, it is almost impossible to analyze every intrinsic connectivity and synaptic interaction based on morphological reconstruction (Fortune and Margoliash, 1995; Foster and Bottjer, 1998; Mooney, 2000; Nixdorf, 1989). However, the synaptic interaction between an isolated neuron pair can be studied by recording the depolarizing or hyperpolarizing membrane voltage response in one cell immediately after the spontaneous or stimulus-evoked spikes from the other cell in the recorded pair (Perkel et al., 1967; Mooney and Prather, 2005; Long et al., 2010).

By blind dual sharp microelectrode recordings from synaptic coupled pairs of an HVC_I_ and an HVC_RA_ neuron, HVC_RA_ axon collaterals often show short-latency, excitatory and strong synaptic connections with interneurons (Mooney and Prather, 2005). A single spike from the HVC_RA_ cell is often sufficient to evoke the HVC_I_ neuron to spike threshold, and spike doublets or triplets from the HVC_RA_ neuron could drive depolarizing responses which can evoke action potentials in the interneuron. Recordings in the same pairs also provide direct evidence that interneurons have synaptic contacts on HVC_RA_ neurons. At the population level, the HVC_RA_ - HVC_I_ coupling is robust and bidirectional, and synaptic transmissions from the interneurons to HVC_RA_ neurons mostly evoke hyperpolarizing responses (IPSPs) in the latter ones (Mooney and Prather, 2005). Bidirectional connections between interneurons and projecting neurons can form bistable networks and generate low-frequency rhythms or no output according to the amount of excitatory input applied to the HVC_RA_ cells (Börgers and Kopell, 2005).

**Figure 4:**
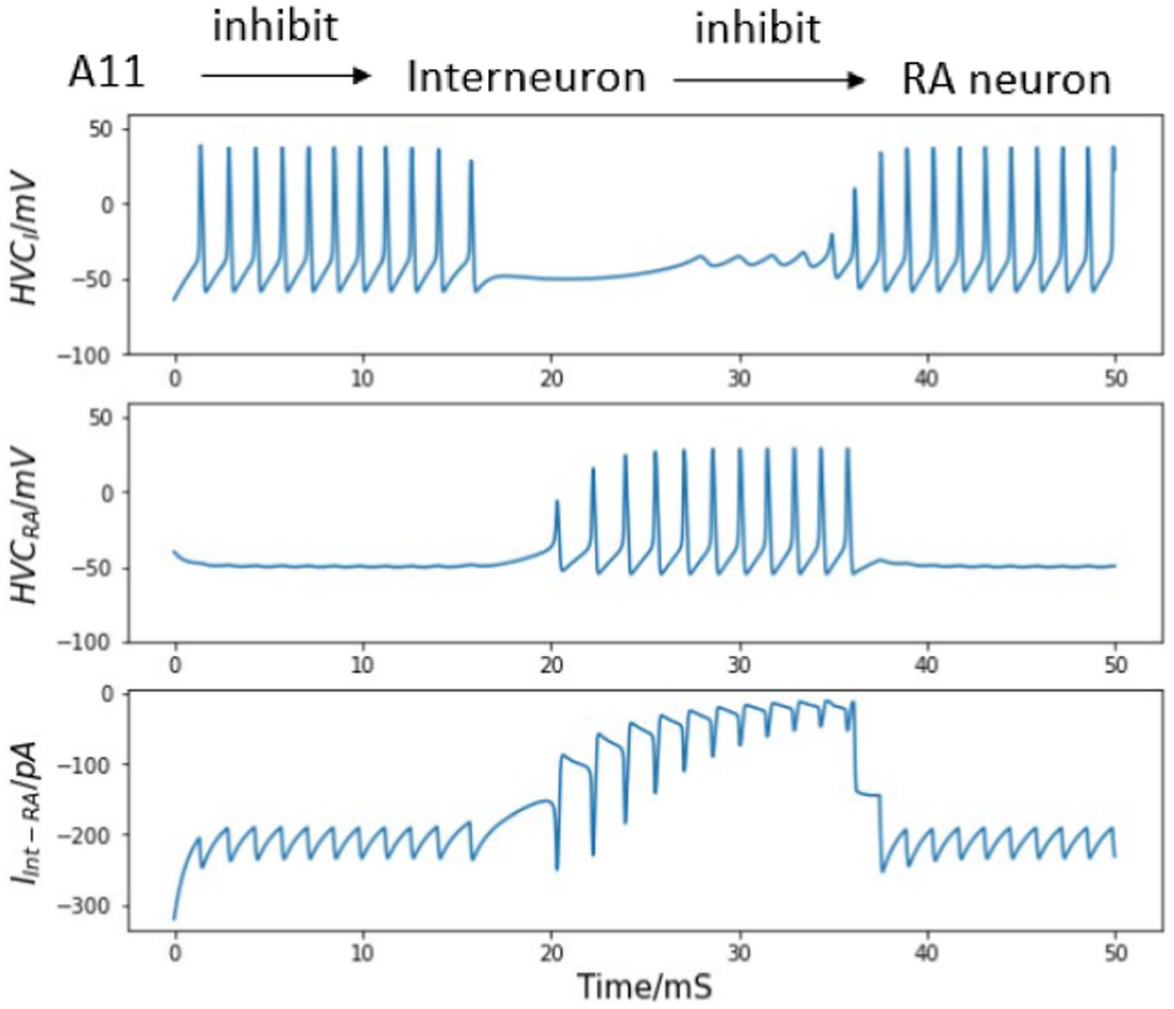
Inhibitory synaptic interaction from HVC_I_ to HVC_RA_ neuron and their voltage traces. Top: diagram of neural circuit simulated in this figure. Note there is only one unidirectional synaptic current between the interneuron and the HVC_RA_ neuron. Top middle: continuous firing of the interneuron and the quiet time induced by trigger current (dynamics of the trigger current is presented in Figure 1). Bottom middle: membrane voltage of the HVC_RA_ neuron with a burst when the interneuron is not active. Bottom: inhibitory current from HVC_I_ to HVC_RA_ cell (Int represents the interneuron, and RA refers to the HVC_RA_ neuron.) See text for important details.

First, we permit one interneuron to form inhibitory synapses directly to an HVC_RA_ neuron. There is no evidence of reciprocal connections from HVC back to A11 cell group, so we only consider the inhibition from A11 axons to HVC_I_ cells. When the trigger input has not arrived and an awake zebra finch is not singing, the population of interneurons are active continually while the HVC_RA_ neurons only stay silent (Kozhevnikov and Fee, 2007). With the synaptic model described in the *Method* section, the inhibitory current from HVC_I_ neuron is strong enough to overcome the background stimulus of 300 pA (Armstrong and Abarbanel, 2016), and silence the HVC_RA_ neuron during the interneuron’s active time (see Figure 4 first 10 ms).

In Figure 4, the simulated interneuron stops firing after the A11 inhibitory current emerges, which enables the HVCRA neuron to generate a burst of spikes. In this context, a burst refers to a series of action potentials which last a very brief time. However, recordings of the HVCRA neuron voltages during singing reveal that a burst usually consists of around 4 spikes and lasts approximately 8 ms (Hahnloser et al., 2002), while the modeled are almost doubled. At this stage the network model output does not fully agree with experimental observations.

**Figure 5:**
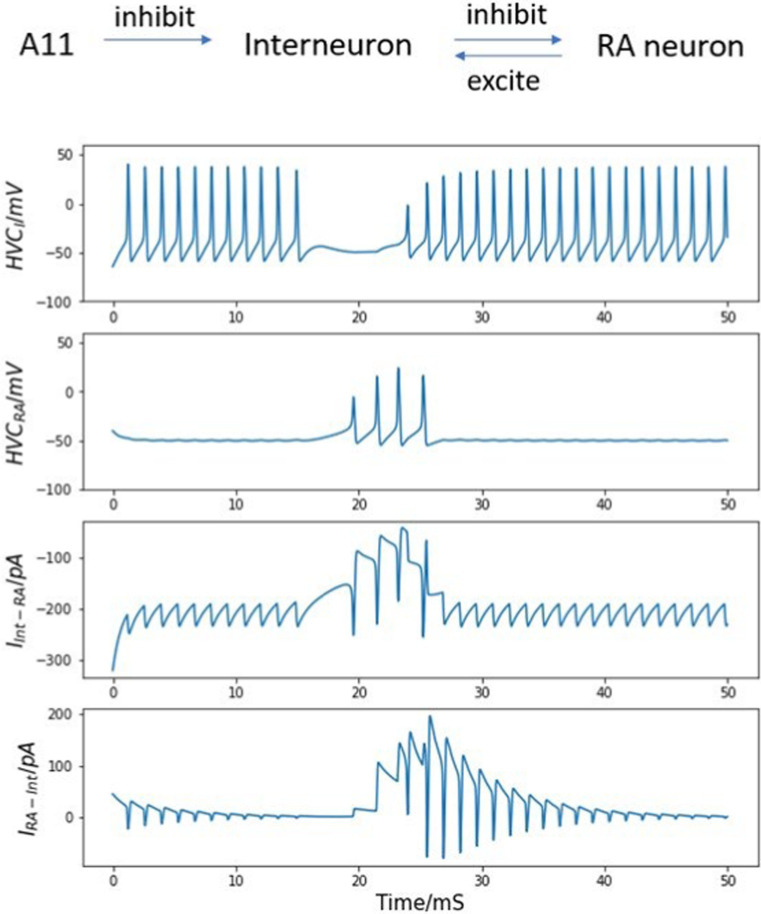
Bidirectional synaptic interactions between an HVC_I_ - HVC_RA_ neuron pair and their voltage traces. Top: synaptic connections among A11 cell group, the interneuron and the HVC_RA_ neuron. Note bidirectional synaptic currents between the interneuron and the HVC_RA_ neuron are included. Top center: continuous firing of the interneuron and the quiet time induced by trigger current. Center: membrane voltage of the HVC_RA_ neuron with a single burst. After involving the reciprocal current from HVC_RA_ neuron back to the interneuron, the burst duration and spike number match experimental observations better than that in Figure 4. Bottom center: inhibitory current from HVC_I_ to HVC_RA_ cell (Int represents the interneuron, and RA refers to the HVC_RA_ neuron). Bottom: excitation connection from HVC_RA_ back to the interneuron. See text for important details.

Second, if the reciprocal excitatory current from the HVC_RA_ neuron to the interneuron is added to the model, the simulated burst behavior better matches the recorded burst pattern of real HVC_RA_ neurons, as illustrated in Figure 5. After the trigger signal appears, the interneuron becomes, leading to the cessation of inhibition HVC_I_ to HVC_RA_ cell, which in turn allows the HVC_RA_ neuron to start its burst. Then, the excitation current generated by the spikes from HVC_RA_ neuron successfully drives the silent interneuron to spike again before the A11 activity completely vanishes. As the HVC_I_ neuron generates continuous spikes again, the interneuron’s sustained firing suppresses the activity of the HVC_RA_ neuron. Now the burst duration and the spike number of the HVC_RA_ neuron closely match the *in vivo* neuron observation. Therefore, the microcircuit model demonstrates that the synaptic interactions of both directions between the interneuron and the HVC_RA_ neuron are necessary for the neural network model to generate the correct activity.

### 3.4 Building a synaptic chain

We now demonstrate how to introduce multiple excitatory neurons to build a complete synaptic chain. A first syllable from the highly stereotyped song motif from the zebra finch is used as an example, and the recorded qualitative behavior of projection neuron populations in HVC during the syllable is reproduced in this process.

**Figure 6:**
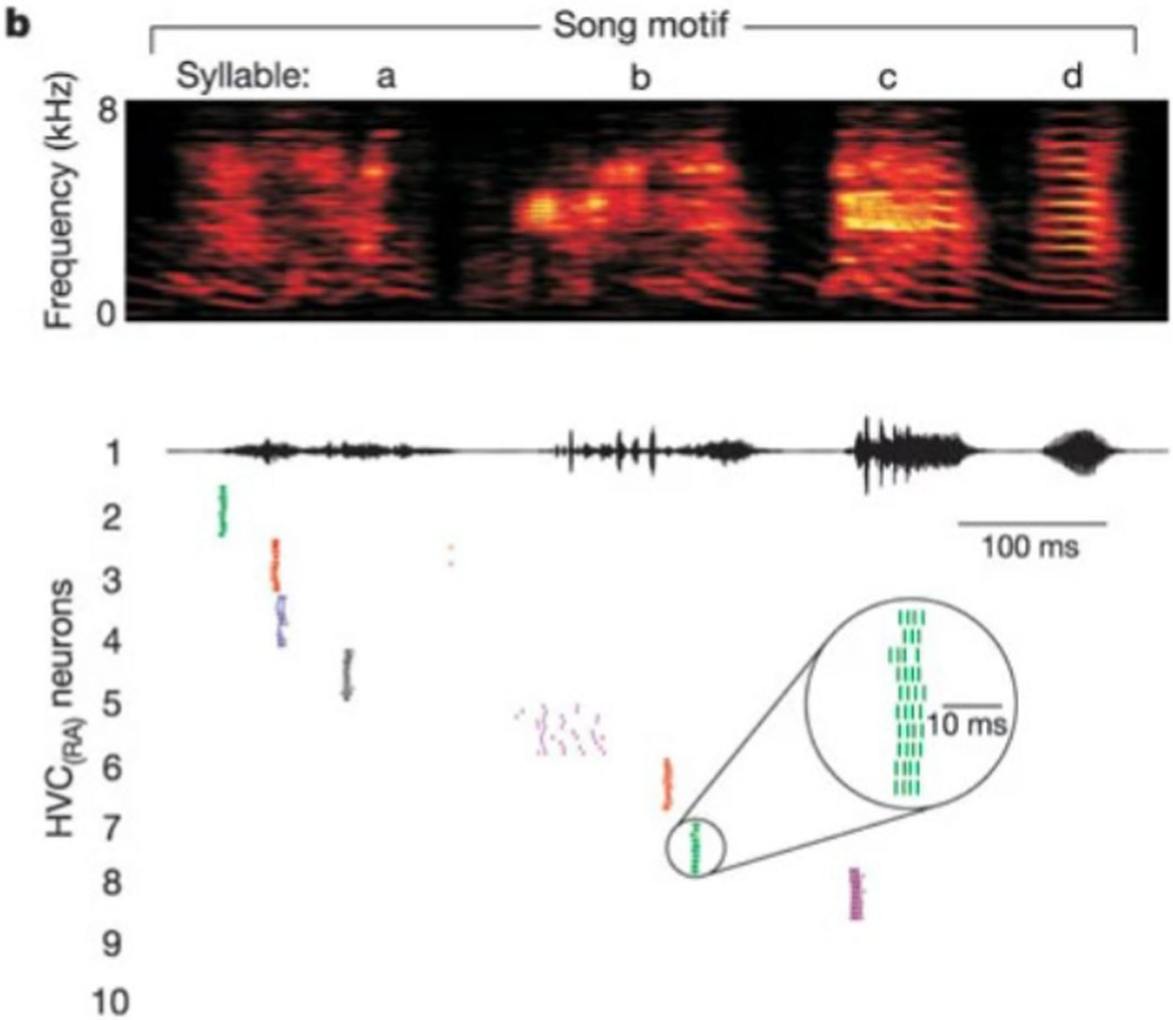
A raster plot of spike times of HVC_RA_ during repeated renditions of the zebra finch motif [Reprinted from Nature by permission from Springer Nature (Hahnloser et al., 2002)]. Readers may find it of interest to compare these spiking times to the voltage plots in Figure 7.

**Figure 7:**
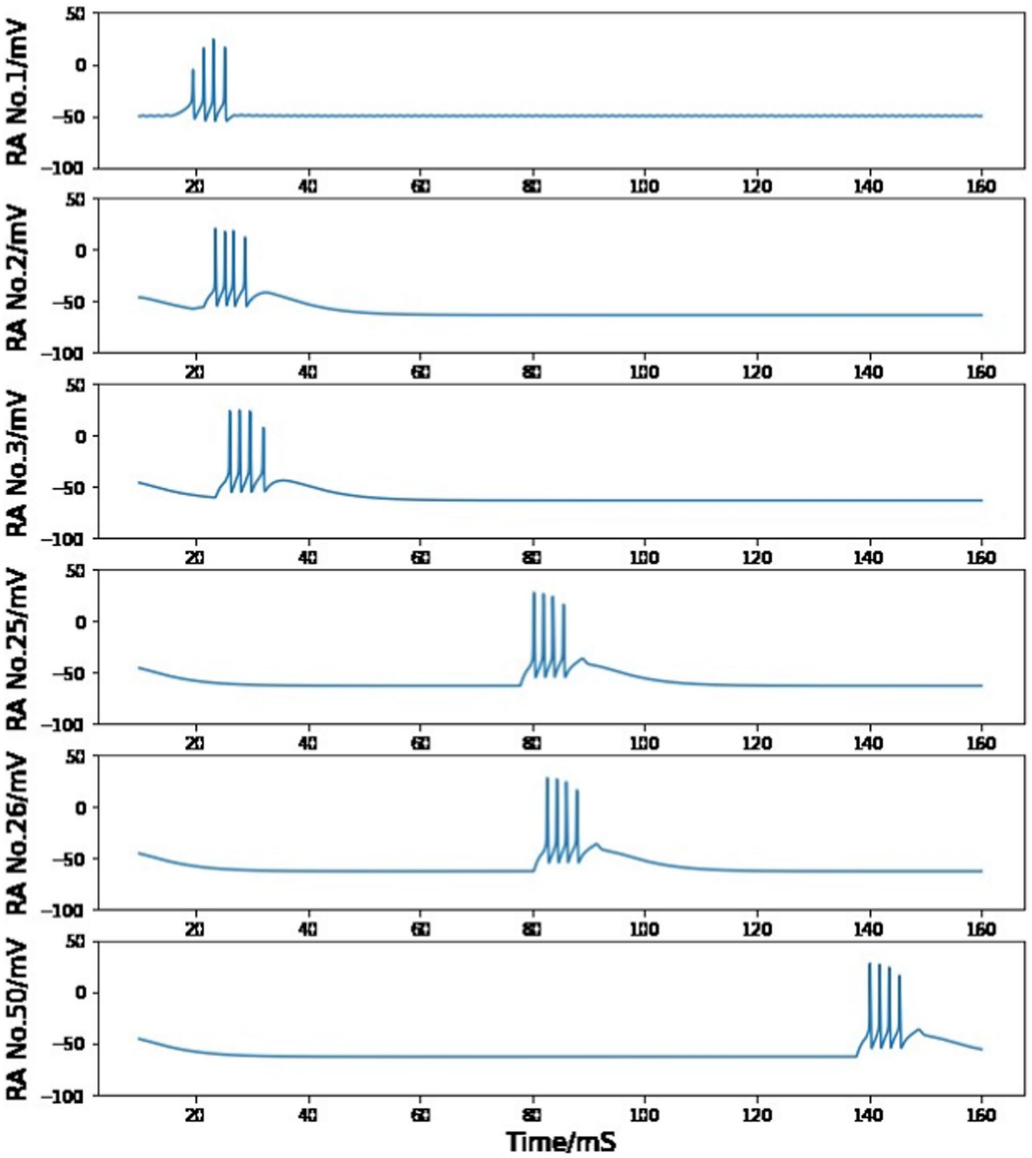
Simulated voltage plots of multiple HVC_RA_ neurons during singing. The first neuron follows the same activity as the HVC_RA_ cell in Figure 5, while this figure shows a time course of 10–160 ms. Given the trigger signal, HVC_RA_ neuron No.2, No.25, No.26, and No.50 reproduce the measured raster plots of neuron 2–5 from Figure 6. Their burst duration, number of spikes in a burst and time intervals between two neuron bursts closely match the experimental recordings.

A full motif contains a fixed number of syllables in an invariant sequence. Although extracellular recordings *in vivo* during singing confirms that each HVC_RA_ neuron usually generates a single burst at a fixed location of one syllable during each song, multiple HVC_RA_ neurons are observed to fire successively. During normal singing, this firing order is fixed, and the time between bursts of two HVC_RA_ cells is also relatively stable. This phenomenon is presented in the experimental raster plot by Hahnloser et al. (2002) in Figure 6, which is compared to our modeling results in Figure 7.

**Figure 8:**
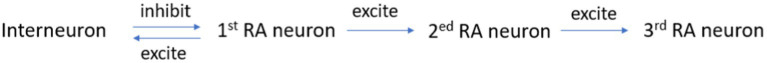
Network architecture enables production of syllables.

This chain-like propagation of spikes among various HVC_RA_ neurons can be explained by direct connections among excitatory neurons (Figure 8). Alternatively, a propagation of silent periods among a sequence of interneurons could occur first, and then the silent time in each interneuron may allow a corresponding HVC_RA_ neuron to burst. The prior mechanism is of higher probability since (1) it agrees with the observed high ratio (about 8:1) of HVC_RA_ to interneuron populations in the nucleus (Armstrong and Abarbanel, 2016), (2) paired recordings show that most HVC_RA_ cell pairs exhibit unidirectional EPSPs, but few homotypic synaptic interactions are observed among interneurons (Mooney and Prather, 2005).

In this network, the first excitatory neuron follows the same HVC_I_ - HVC_RA_ neuron interaction and the voltage trace in Figure 5, and passes that burst to the second HVC_RA_ neuron by homotypic excitation current, and so on (Figure 8). Most HVC_RA_ neurons in the chain (except for the first HVC_RA_ neuron) do not fire spontaneously considering the general inhibitory effect from the HVC_I_ and HVC_X_ projecting neuron populations. Simulating the potential inhibition current from each individual neuron is beyond the scope of this paper, but we account for this phenomenon by lowering the background stimulation to 50 pA, which is known to allow those excitatory neurons to stay silent during *in vitro* experiments (Daou et al., 2013). The average maximal conductance of excitatory synaptic currents between two neurons is estimated to be around 7 nS, as stated in the *Method* section. However, if the synaptic connection strength for homotypic HVC_RA_ cell pairs is set to 7 nS, the excitatory input would not be large enough to awake an HVC_RA_ neuron (see the *Model Robustness* section for further discussions about tuning this parameter values). Therefore, the synaptic connection strength for homotypic HVC_RA_ cell pairs is set to 8.2 nS to ensure that the postsynaptic neuron will copy the burst pattern of the presynaptic neuron. The only exception happens at the first HVC_RA_ cell which is directly impacted by the trigger signal. The spikes in its one-time burst are relatively weak, so the value of g_RA,RA_ for the first and second neuron is tuned to 10 nS so that the second HVC_RA_ neuron can generate the same number of spikes.

Figure 7 shows the simulated cross-membrane voltages of the sequentially connected excitatory neural network in response to the neurotransmitter trigger signal depicted in Figure 1. The firing timings of neuron No.2, No.25, No.26, and No.50 correspond closely with the repeated electrode recordings of neuron 2–5 in the plot of Hahnloser et al. (2002) (Figure 6). During the first syllable, each HVC_RA_ cell generates a short burst consisting of four spikes. The time span of a single burst is on the order of 10 ms, and the short (∼ 3 ms) or long (∼ 50 ms) time intervals between spikes from different neurons are also reproduced in Figure 7.

### 3.5 Model robustness

**Figure 9:**
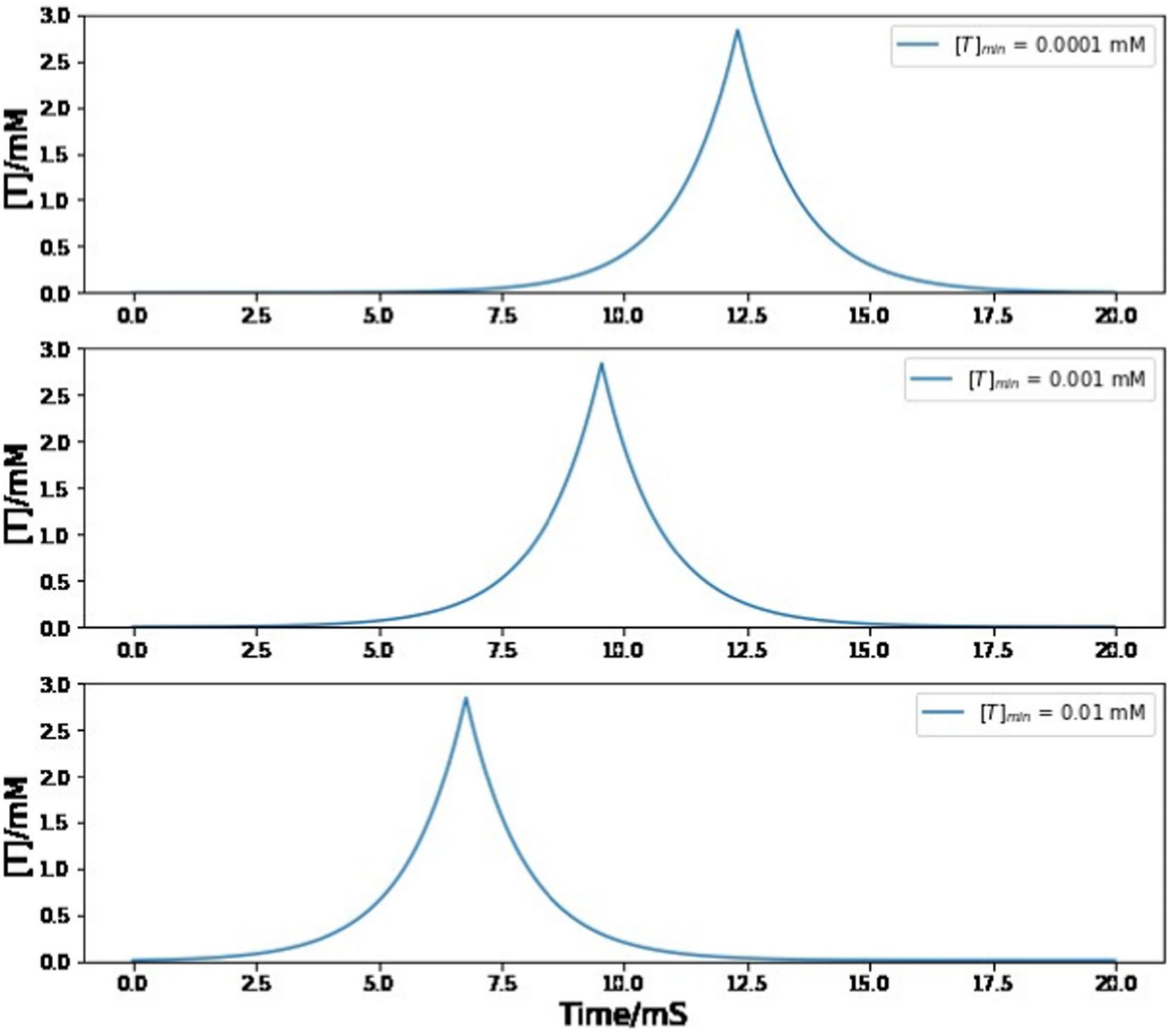
Simulated trajectory of neurotransmitter concentration in the synaptic cleft between A11 axons and HVC neurons under different choices of [T]_min_ value. Varying the value of [T]_min_ does not affect the magnitude, duration, and shape of neurotransmitter dynamics. The behavior of the neural network model exhibits considerable robustness with respect to variations in the value of [T]_min_.

In our numerical simulations, most parameter values are obtained from published literatures, with two exceptions: [T]_min_ and g_RA,RA_. There is no convincing analysis of the baseline concentration of neurotransmitters before the onset of a trigger signal, so we choose the [T]_min_ value to be 0.001 mM, which is much smaller than the maximum neurotransmitter concentration [T]_max_. Fortunately, varying the value of the minimum neurotransmitter concentration does not change the simulation result as long as it stays positive and small compared to [T]_max_. In Figure 9, even if the value of [T]_min_ increases/decreases by 10 times, the magnitude, duration, and shape of neurotransmitter dynamics stays almost the same. The only difference that is introduced by the [T]_min_ value is the peak time of the neurotransmitter concentration from A11 axons, which has no impact on any simulation conclusions since this paper does not focus on the exact onset time of the trigger current.

**Figure 10:**
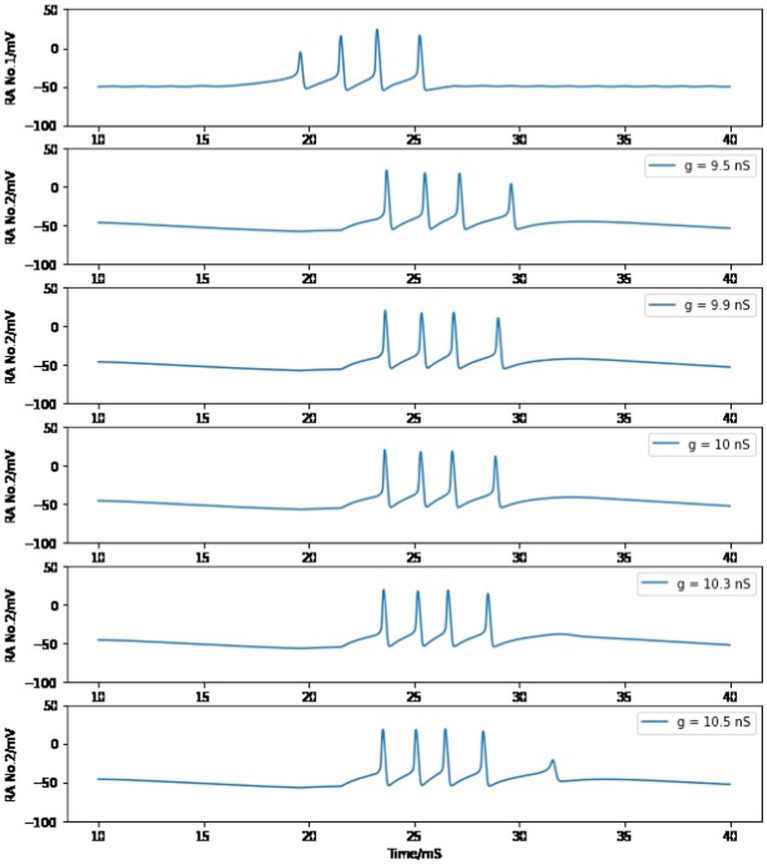
Simulated voltage plots of the first HVC_RA_ neuron and the second HVC_RA_ neuron with different values of maximum conductance for the synaptic interaction between them. See text for more information.

As stated in the section *Building a syllable*, the value of maximum conductance for connecting the chain of HVC_RA_ neurons is chosen to be 8.2 nS or 10 nS for the first pair of HVC_RA_ neurons, which allows the postsynaptic cell to reproduce the burst duration and spike number of the presynaptic neuron. As discussed in the *Method* section, the maximal conductance of AMPA-mediated current for a single synaptic connection is measured between 0.35–1.0 nS, and there are approximately 10 synapses between a pair of connected neurons. Therefore, a reasonable value of maximum conductance should be in the range of 3.5–10 nS, which includes our proposed parameter value. Furthermore, the presynaptic HVC_RA_ neuron will still pass its firing pattern to the postsynaptic cell if this maximum conductance varies a small portion. When the first HVC_RA_ neuron is the presynaptic cell, g_RA,RA_ is tuned to a larger value compared to other interactions since this neuron’s first burst spike is weaker than full firing. As long as g_RA,RA_ stays within the range of 9.9–10.3 nS, the second HVC_RA_ neuron will still generate four full spikes. Otherwise, the postsynaptic neuron burst will not reach four full firings if the maximum conductance is too small, or there will be a fifth miniature peak if the value is too large (See Figure 10). We select 10 nS as the modeling parameter value because it is within the reasonable value of measured maximum conductance between two neurons.

**Figure 11:**
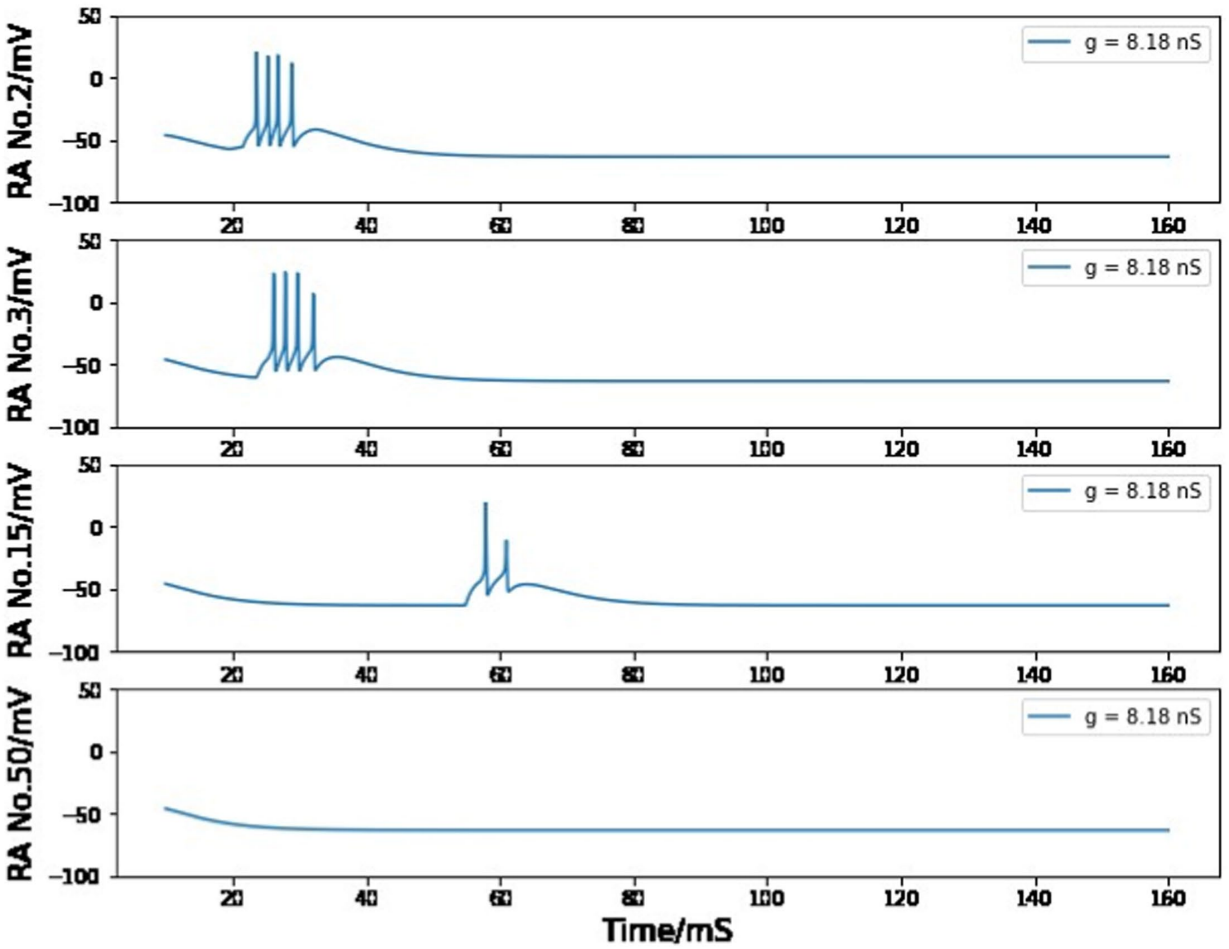
Simulated voltage plots of multiple HVC_RA_ neurons during singing with g_RA,RA_ = 8.18 nS. For the first several neurons, the postsynaptic cell is able to copy the burst behavior of the presynaptic neuron, but this one-time burst gradually disappears as it is passed through more synaptic connections.

**Figure 12:**
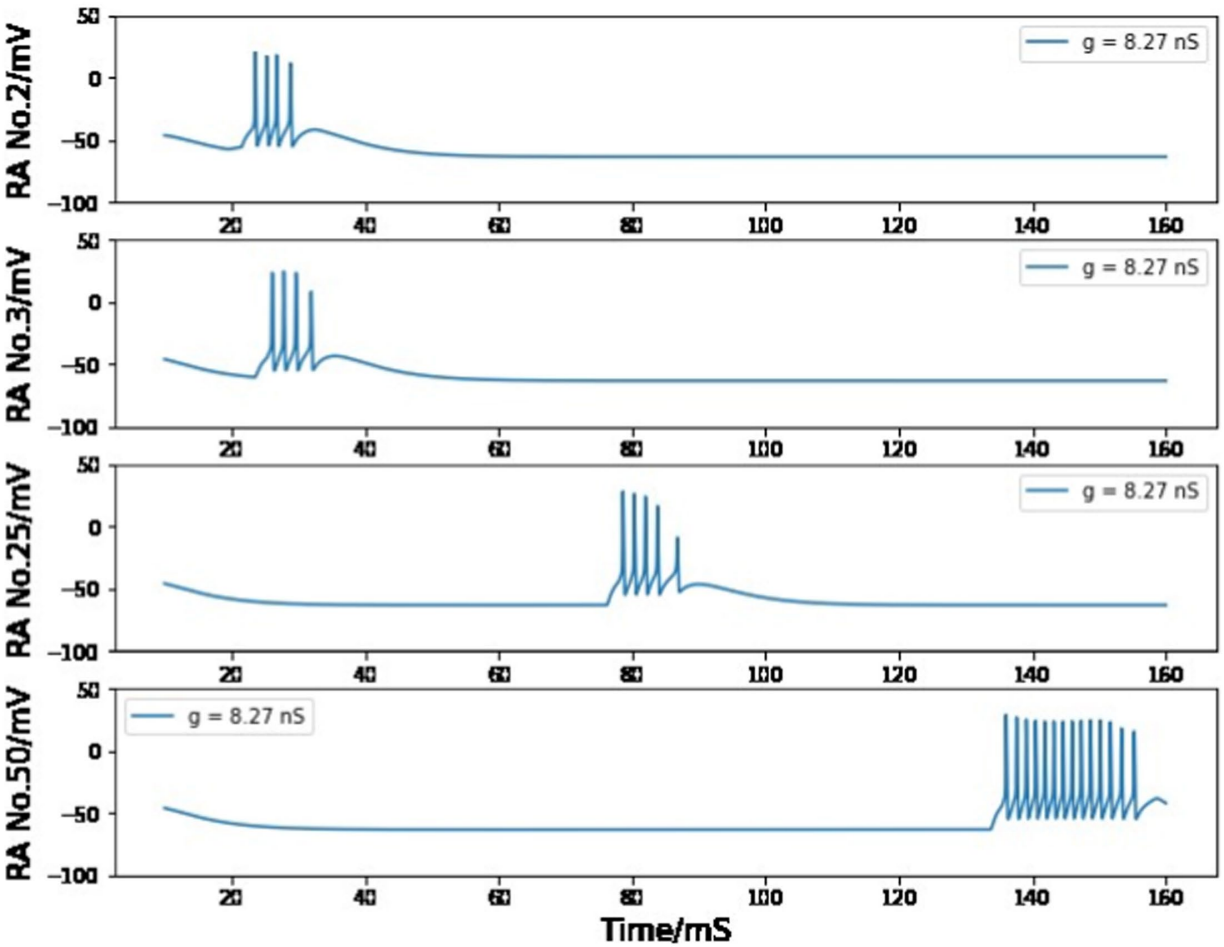
Simulated voltage plots of multiple HVC_RA_ neurons during singing with g_RA,RA_ = 8.27 nS. For the first several neurons, the postsynaptic cell is able to copy the burst behavior of the presynaptic neuron, but more spikes are added to the burst because of the strong synaptic interaction strength.

In the chain of HVC_RA_ neurons after the first pair, the maximum conductance is set to 8.2 nS to ensure that the burst of four spikes can be spread by the unidirectional connections. If the value of g_RA,RA_ is smaller than 8.18 nS, the burst will gradually disappear during this long transfer process (Figure 11); if it is larger than 8.27 nS, the burst spike number will increase as more neurons are added to this sequence of HVC_RA_ neurons (Figure 12). The selected value of 8.2 nS for the maximum conductance in the sequence of HVC_RA_ neurons is reasonably close to the measured median value of maximum conductance for excitatory currents, which is 7 nS.

**Figure 13:**
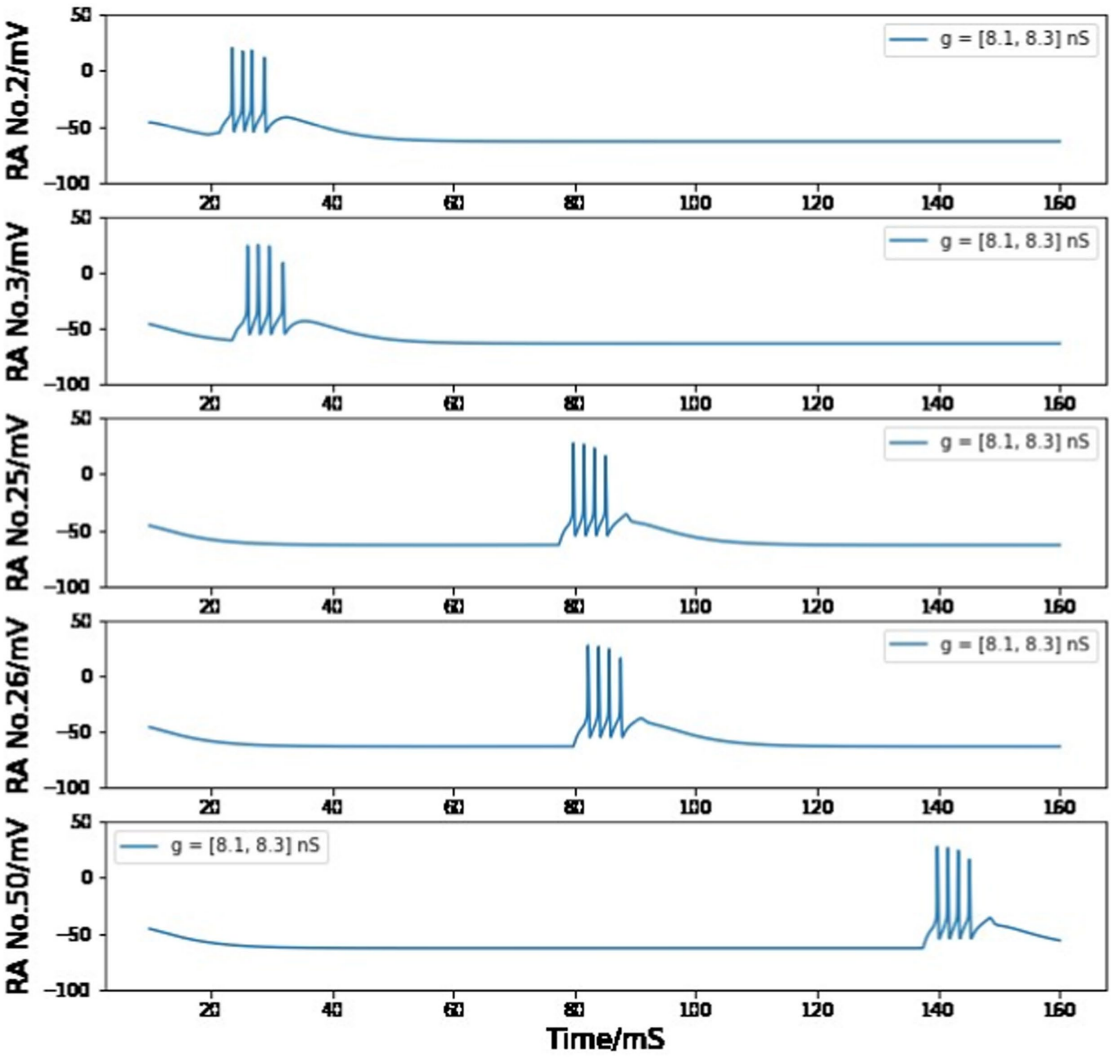
Simulated voltage plots of multiple HVC_RA_ neurons during singing as the value of g_RA,RA_ for each synaptic interaction is evenly distributed in the range of 8.1–8.3 nS. The firing patterns of all neurons are similar to Figure 7 from *Building a syllable* section.

However, if we only care about one pair of HVC_RA_ neurons, the voltage trace of the postsynaptic cell will still be very similar to the presynaptic one when g_RA,RA_ = stays in the range of 8.1–8.3 nS. Moreover, if the maximum conductance value of each HVC_RA_ - HVC_RA_ neuron pair is distributed uniformly between 8.1 nS and 8.3 nS, the neuron behavior and model conclusion will not be changed (see Figure 13).

## 4 Discussion

This paper has described a HVC neural network model consisting of single neuron models of HVC_I_ and HVC_RA_ neurons in the HVC nucleus, as well as synaptic current equations and a trigger signal model based on Ben-Tov et al. (2023). We began with single neuron models describing fundamental ion channels in the HVC_RA_ and HVC_I_ neurons, and showed that the HVC_RA_ neuron fired continuously under a background current above its experimental threshold. After introducing an inhibitory connection from the interneuron to the HVC_RA_ neuron, this HVC_RA_ neuron became silent, which was expected in the absence of singing behavior. Then, the current from A11 cell group to HVC allows the interneuron model to reproduce the continuous firing with intermittent pauses reminiscent of the HVC_RA_ neurons during song production. Now, the HVC_RA_ neuron was able to generate a burst, but the burst duration and spike quantity initially did not match experimental observations. This mismatch was resolved after the excitatory interaction from the HVC_RA_ neuron back to the interneuron was added to the circuit, which suggested that the bidirectional connections in the HVC_RA_-HVC_I_ neuron pair observed *in vitro* might be necessary to maintain the firing pattern of neurons in this nucleus. Finally, more HVC_RA_ neurons were included in a chain configuration, successfully reproducing the time-locked sequential burst from multiple HVC_RA_ neurons during a syllable. All but one of the parameters in the single neuron models and synaptic current models were backed by other simulation or experimental papers. The only parameter that was fine-tuned was the maximal conductance of synaptic current between two HVC_RA_ neurons. In the *Results* section, we discussed the possible range for the parameter and showed that the fine-tuned value fell within the measured range of maximal conductances for excitatory synaptic currents.

The model could be further applied to describing the functions and dynamics of HVC neurons in other songbirds such as Bengalese Finch or canary (Clayton, 1987; Brenowitz et al., 1997). The fine-tuned parameter in the synaptic current model may also provide a reference for synaptic coupling strength in the avian brain. Our work in this paper offers tools to understand the dynamics of HVC and its function as a song motor in the avian song system.

### 4.1 Another type of projecting neurons

Our model focuses exclusively on HVC_RA_ projecting neurons as well as interneurons which serve as an important coordination for projecting neurons to function properly. This network does not include HVC_X_ projecting neurons, a third major type of neurons in this region. HVC_X_ neurons projects onto area X, which in turn give rise to the anterior forebrain pathway (Mooney, 2009). The synaptic connections from HVC_X_ to HVC_RA_ neurons are detected but relatively less frequently than the connections from HVC_I_ to HVC_RA_ neurons. The chance that the spike-evoked responses from HVC_X_ to HVC_RA_ cells are hyperpolarizing or depolarizing are approximately the same (Mooney, 2009). Moreover, induced death of HVC_X_ neurons does not significantly alter neuronal recruitment or song productions in adult zebra finches (Scharff et al., 2000). Therefore, the role of HVC_X_ neurons in coordinating HVC_RA_ neuron behavior may be not as critical as interneurons, and it is beyond the scope of this paper to address alternative detailed network structures involving all three major populations of neurons, resulting in the requirement for further observations and studies.

### 4.2 Possible additional ion currents

Previous studies have proposed several models of individual HVC_RA_ neurons and interneurons. These models contains different combinations of ion currents, as well as different equations and parameters for each ion current (Jin et al., 2007; Daou et al., 2013; Breen et al., 2016; Kadakia et al., 2016; Armstrong and Abarbanel, 2016). Our single neuron models are adapted from earlier works. Each neuron model in this paper includes only the basic ion channels which previous papers agree to be important for that specific neuron type. Follow-up work may examine additional possible ion currents such as A-type potassium current, high-threshold L-type calcium current, persistent sodium current, and calcium dependent potassium current.

### 4.3 Previous models of HVC sequence generation

In a previous model of sequence generation by HVC_RA_ neurons, Jin proposed that the burst sequence is generated by a synburst chain within the HVC_RA_ population alone (Jin, 2007). The model assumes that HVC_RA_ neurons are intrinsically bursting, and the burst durations are set by cellular properties. Burst sequences generated from the model are similar to those observed in HVC. However, the paper assumes that the burst sequences are not driven by input from any upstream brain areas, and it does not address how to initiate the spiking activities in the chain of neurons. The paper claims that its intrinsic bursting model improves the spike robustness against synaptic connectivity strength. However, most parameters proposed in this neural network model do not have experimental or simulation evidence to validate their plausibility, and the improvement of robustness has only been tested based on the proposed group of parameters. The model also neglects the influence of interneurons on the HVC_RA_ population.

Cannon et al. (2015) describes a feedforward excitatory chain model with local feedback inhibition, designed to generate stereotyped neural sequences. The model integrates inhibition into the series propagation of HVC_RA_ neuron activations, but the proposed integration mechanism is carefully engineered without biophysical motivations. The individual neurons are modeled using quadratic integrate-and-fire equations. The excitatory and inhibitory post-synaptic current equations are independent of pre-synaptic voltages. The paper does not intend to describe HVC neurons and their connections in biological details, so most parameter values employed in this model lack experimental validation and are chosen primarily to ensure the functionality of the model.

A more recent model reproduces the observed series of HVC_RA_ activities by introducing a small neuronal loop capable of transitioning between an “active” and “quiescence” state (Armstrong and Abarbanel, 2016). Multiple neural loops are arrayed in a chain, stimulated in sequence to excite an “active” state that propagates down the chain. The mechanism of connectivity between two neuronal loops and the method of achieving a sequence of ‘active’ states is unspecified. Certain parameter values lack experimental or simulation evidence to support their reasonability, and the sensitivity of the modeling results upon those parameter values has not been examined.

### 4.4 Building a complete song

In this paper we consider what happens when the neural network is exposed to a neurotransmitter pulse induced by a male zebra finch’s need to attract a female, and the injected neurotransmitters start the first syllable of a motif. It is interesting to further examine the plausibility of generating a complete song following similar neuromodulator mechanisms. Each bird’s whole song comprises an average of 12 harmonic syllables of around 80–200 ms each in duration (Woolley et al., 2010; Glaze and Troyer, 2016). Within our framework, the full motif could be explained by a chain-like propagation linking HVC_RA_ to HVC_RA_ neurons, similar to how to construct the first syllable. This continuous synaptic architecture within HVC agrees with the observation that local HVC circuit connectivity contains sufficient information to propagate throughout the song sequence during sleep replay (Elmaleh et al., 2021). Since HVC is responsible for temporal order rather than sound of syllables (Fee and Scharff, 2010, Long and Fee, 2008; Simpson and Vicario, 1990), we do not worry about how to generate acoustic features for different syllables.

An alternative scenario would be that the active series of syllables is achieved by sequentially arrived neuromodulator from A11 axons. Even though the excitatory synaptic connections between HVC_RA_ neurons simulate the distributed bursts inside one syllable, it is possible that each syllable represents a relatively independent structure in the nucleus. During experiments of singing interruption, individual syllables are more robust than the full song: direct electrical interference is necessary to interrupt a syllable, but ongoing motif can be interrupted by noninvasive techniques such as strobe light (Cynx, 1990; Armstrong and Abarbanel, 2016). Experimental results also show that the thalamic axon activity is critical for starting the following syllable but no for completing the ongoing syllable (Moll et al., 2023). These evidences suggest that the connectivity among syllables may follow a different mechanism from the direct synaptic interactions. We speculate that a neural feedback loop involving other nucleus may activate a succession release of neurotransmitters, which triggers multiple syllables to play a whole motif.

Another alternative to achieve a full song would be that the neurotransmitters diffuse and arrive at different parts of HVC sequentially. In this case, the microcircuits of neurons responsible for their own syllables are located at different locations throughout the nucleus. The triggering neurotransmitters are released from the A11 axons all at once and then diffuse within HVC, activating spatial organized microcircuits to sing each syllable sequentially.

### 4.5 Learning

Multiple HVC neurons of different types form correlational connectivity to ensure the functionality of the nucleus. How could neurons in junior zebra finches develop this cooperation during learning? One possibility is that these neurons adapt both spatial organization and synaptic plasticity to achieve bird’s own song. There is evidence of directed neural networks within the HVC matures during sensorimotor learning (Day et al., 2013), which indicates the existence of spatial organization development. Neurons in the HVC also regulate their ion channel conductances over the arc of development (Daou and Margoliash, 2020), suggesting that the strength of synaptic currents may also covary during vocalization development.

## Data Availability

The raw data supporting the conclusions of this article will be made available by the authors, without undue reservation.
